# Predictors of Mortality in Mechanically Ventilated Critical Pertussis in a low Income Country

**DOI:** 10.4084/MJHID.2014.059

**Published:** 2014-09-01

**Authors:** Aida Borgi, Khaled Menif, Sarra Belhadj, Narjess Ghali, Loukil Salmen, Asma Hamdi, Ammar Khaldi, Aida Bouaffsoun, Sonia Kechaou, Amel Kechrid, Asma Bouziri, Nejla Benjaballah

**Affiliations:** 1 Pediatric intensive care unit, children’s hospital Bechir Hamza of Tunis; 2 Microbiology laboratory, children’s hospital Bechir Hamza of Tunis; 3 Group of Bioinformatics and Mathematical Modeling, Institute Pasteur of Tunis

## Abstract

**Background:**

Critical pertussis is characterized by severe respiratory failure, important leukocytosis, pulmonary hypertension, septic shock and encephalopathy.

**Aim:**

To describe the clinical course of critical pertussis, and identify predictors of death at the time of presentation for medical care.

**Methodology:**

Retrospective study conducted in children’s hospital Tunisian PICU between 01 January and 31 October 2013. Patients with critical pertussis confirmed by RT-PCR and requiring mechanical ventilation were included. Predictors of death were studied.

**Results:**

A total of 17 patients was studied. Median age was 50 days. Mortality was 23%. Predictors risk of mortality were : high PRISM score (Pediatric Risk of Mortality Score) (p=0,007), shock (p=0,002), tachycardia (p=0,005), seizures (p=0,006), altered mental status (p=0,006), elevated WBC count (p=0,003) and hemodynamic support (p=0022). However, the difference did not reach statistical significance in comorbidity, pneumoniae, high pulmonary hypertension or exchange transfusion. Concomitant viral or bacterial co-infection was not related to poor outcome.

**Conclusion:**

Young infants are at high risk to have critical pertussis. Despite advances in life support and the treatment of organ failure in childhood critical illness, critical pertussis remains difficult to treat.

## Introduction

Pertussis is an acute respiratory illness caused by *Bordetella pertussis* (B. pertussis). Critical pertussis (CP) is defined as pertussis disease that results in pediatric intensive care unit (PICU) admission or death. It is characterized by severe respiratory failure, important leukocytosis, pulmonary hypertension, septic shock and encephalopathy. Despite intensive care management, it causes substantial morbidity and mortality for children especially among young infants. Resurgence of pertussis in the last 20 years is evident from the Centers for Disease Control (CDC).^1^ Several reasons for this resurgence have been proposed, including genetic changes in Bordetella pertussis, lessened potency of pertussis vaccines, waning of vaccine-induced immunity, greater awareness of pertussis, and the general availability of better laboratory tests.^2^ A new resurgence was seen in 2013 in Tunisia even in the presence on a high (98%) coverage of childhood vaccination.^3^ The purpose of this study was to describe the institutional experience in the management of infants with CP admitted in year 2013 at Children’s hospital Bechir Hamza of Tunis, reporting the relationship between method of presentation, therapies and outcome in order to identify factors associated with death.

## Patients and Methods

### Setting

The study was conducted in the PICU of Children’s Hospital Béchir Hamza of Tunis. The PICU is in a university-affiliated children’s hospital and provides intensive care services to a national pediatric population of 850 000 children less than 15 years old. The hospital has 360 beds, and the PICU has 16 beds (500 admissions/year).

### Data collection

Since 2009, real-time reverse transcriptase–polymerase chain reaction (RT-PCR) for detection of *Bordetella pertussis* was performed on nasopharyngeal and/or tracheal secretions for all patients admitted with suspected pertussis or bronchioltis.

Suspected pertussis symptoms were consistent with the World Health Organization (WHO) definition case.^4^

Nasopharyngeal secretions (NPS) obtained by aspiration on sterile tubes were the specimens of choice for Bordetella detection by culture and/or quantitative real-time polymerase chain reaction (qPCR). Tracheal samplings were also accepted. Time from specimen collection to receipt in the laboratory was 20 min to 2 days, with no transport additives. The RT-PCR targets include the IS481, commonly found in B. pertussis, B. bronchiseptica, and B. holmesii; the IS1001, specific of B. parapertussis, in combination with the pertussis toxin promoter region gene (ptx) of B. pertussis; and the recA gene-specific of B. holmesii. NPS and/or tracheal sampling for viral detection through RT-PCR were also performed in all infants. We reviewed the medical charts of 17 children [median age 56 days (range 24–630); sex-ratio: 0.88] admitted to our PICU between 01 January to 31 October 2013 for confirmed CP by RT-PCR. Only mechanically ventilated patients were included.

Clinical data included: patient demographics, neonatal history, immunization status, presentation. Routine laboratory data and chest radiographs were also performed at admission in order to reveal infiltrate or consolidation imagings. Severity of Pediatric Risk of Mortality Score (PRISM) was evaluated at the time of PICU admission. During the hospitalization, the presence of complications and coinfections with viral or bacterial pathogens were checked, and all therapeutic interventions, including duration of respiratory or circulatory support with or without sedation, muscle paralysis, inhalation of nitric oxide were recorded.

Extracorporeal Membrane Oxigenation (ECMO) treatment was not available in Children’s Hospital Béchir Hamza PICU.

Based on the outcome, we divided subjects on two groups “survival” and “deaths” and we studied risk factors for poor outcome.

### Statistics

Data analysis was performed using SPSS (version 17.0). Mann-Whitney test, Fisher exact test were applied as appropriate. A 2-sided *P* < 0.05 was considered significant.

## Results

**Figure 1:**
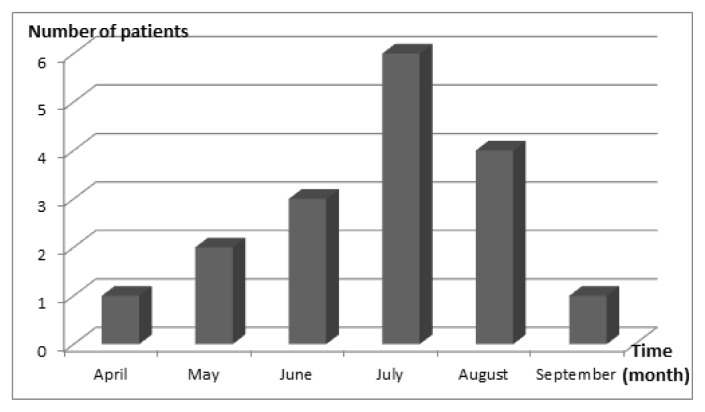
Number of patients admitted for critical pertussis and month distribution

A total of 17 children were included. Fifty two per cent of the enrolled patients were female. Demographics and illness history are summarized in table 1. Median age at admission was 56 days (range 24–630) and nine patients were under 2 months of age. Month distribution is displayed in figure 1. Median PRISM was 12 (IQR 4–27). Twelve patients (71%) were unimmunized. Five had received only one dose of pertussis vaccine and were partially immunized. The source of infection was identified in 9 (53%) cases. Mother-to-infant transmission occurred in six cases (66%).

Clinical presentation on admission showed repeated cough with desaturation and bradycardia in 15 patients (88%), apnea in 1 patient (6%) and hypoxia in 8 patients (47%). Six patients (35%) had no respiratory distress when coughing stopped. Tachycardia more than 200 bpm was present in five cases (29%), shock in 4 cases (23.5%), seizures in 6 cases (35%), and altered mental states in 3 cases (17.6%). Among patients where an echocardiogram was done, 2 patients had pulmonary hypertension. Echocardiography was done on 4 patients.

Median initial WBC was 41 × 109 L–1 (IQR 3.8–125.10^9^). Initial WBC counts greater than 100 × 10^9^ L–1 was reported in all deaths. Chest radiograph on admission identified pneumonia in 14 patients (82%) and was normal in only 3 cases(17.6%). Based on blood gases, seven patients had PaO2/ FiO2 <200.

Viral studies were available in 15 subjects (88%). Eight patients (53%) had viral coinfection with *Rhinovirus* in 7 cases and with R*hinovirus and Coronavirus* in only one case. Two among 4 deaths had coinfection with rhinovirus. Viral coinfection was not related to mortality in our study.

All patients required mechanical ventilation. Intubation was done immediately at PICU admission for 15 cases and after a median delay of 2 days of non invasive ventilation for the others. The median duration of ventilation in survivors was 9 days (IQR, 3–20 days).

The indications for intubation were: cyanogenic cough in 11 patients (64%), severe hypoxic respiratory failure in 4 cases (23%), septic shock in one case (6%) and encephalopathy in the last case (6%). High frequency ventilation was performed in one patient (6%). All patients received fentanyl and midazolam sedation and only 2 patients (12%) were treated with muscles paralysis.

Four patients (23 %) received inotropes and 2 (11.7 %) nitric oxide. Five patients (29%) received leukoreduction therapy by the mean of exchange transfusion. The median WBC value was 105 ×10^9^ L–1 (IQR 71–125) prior to leukoreduction. The WBC deceased to a half in all patients after blood exchange [median WBC=46 ×L–1 (IQR 45–60)].

All patients received intra venous erythromycin to reduce their infectivity. Four patients (23%) had bacterial coinfection with bacterial proof in 3 cases (*enterobacter cloacae* in 1 case, *streptococcus pneumoniae* in one case and *hemophilus influenzea* in the last case). Bacterial coinfection occurred in two deaths. The cause of death was related to septic shock in one of them.

Complications occurred in 6 patients: nosocomial infection in 5 patients and femoral venous thrombosis post catheterization in one case.

Four patients (23%) died during the acute hospital course; 2 of these deaths occurred few hours after PICU admission. Time of death ranged from 1 to 30 days after admission. The death was attributed to refractory shock in 3 cases and cerebral death in 1 case. High PRISM (p=0,007), shock (p=0,002), tachycardia (p=0,005), seizures (p=0,006), altered mental status (p=0,006), elevated WBC count (p=0,003) and hemodynamic support (p=0,022) significantly differentiated patients who died from survivors.

## Discussion

Pertussis remains a public health worldwide problem even where coverage for childhood vaccination is high.^5^ Its prevalence in Tunisia was 20% between 2007 and 2011.^3^ There was a significant increase in 2013. The majority of cases were admitted in July. Occurrence in summer showed that pertussis did not mimick bronchiolitis period. Concomitant viral infection was observed in 8 cases. Rhinovirus was the most isolated viruses. Co-respiratory viral infections are common with pertussis. The most frequently identified agents are rhinovirus and respiratory syncytial viru**s** ().^6^,^7^ To our knowledge, it is the first report of rhinovirus infection in the summer. Partial immunization appeared to be a risk factor of mortality but infants less 2 months old have no maternal antibodies neither active immunization. The age of infants seemed to be a confounding factor. So that, we have completed the statistical analysis by adjusting for age, and we have found that the mortality is not significantly different between immunized babies and unimmunized ones (p = 0.142).

In our study, we observed mortality rate of 23%. The mortality reported in the literature was less than 10%.^8^ In these studies, they included all pertussis PICU admission. We excluded all patients who do not need mechanical ventilation. Pierce C et al^9^ reported a series of 13 critically ill infants with pertussis and the mortality rate was 31%.

A recently published study summarized experience with infants admitted with pertussis to an Australian PICU over a 20-year period.^10^ The authors reported that infants dying of pertussis suffered from severe pneumonia, circulatory failure, encephalopathy, and multiple organ system failure. Mortality in malignant pertussis was 70% and significantly higher in infants who were younger than 6 weeks (84%).^11^ Median age for deaths in our study was 88 days.

The predictors of death, reported in the literature included young age, lack of and/or incomplete vaccination, pneumonia, seizures, severe leukocytosis, high pulmonary hypertension, the need for circulatory support.^12^ High pulmonary hypertension did not influence mortality in our study; however, echocardiography was recorded in only 4 among 17 patients. Our study had some limitations: the small size of our cohort and retrospective review. In conclusion, studies on clinical courses of children with critical pertussis like those admitted to the PICU are scarce in the literature; most of the published studies were done in a single centre, furthermore were retrospective cohort studies having a small sample size**.**^13^ Despite advances in life support and the treatment of organ failure in childhood in critical care units, pertussis remains difficult to treat. Exchange transfusion and leukapheresis have been reported effective in small cohorts for correcting the pulmonary hypertension that characterizes critical pertussis, and facilitating the oxygenation.^14^,^15^ We performed exchange transfusion in 5 patients with leucostasis more than 100.000 WB count. Three of them died even if exchange transfusion was effective in rapidly dropping the numbers of circulating WBCs. The death occurred at 30 days of PICU stay from encephalopathy. The was also a death for septic shock, related to untreated bacterial co-infection in forth day on admission. It is possible that ECMO could have improved prognosis of children with refractory septic shock,^16^ but this treatment was not available in our country.

## Conclusion

Young infants are at high risk of critical pertussis. Despite advances in life support and the treatment of organ failure in childhood critical illness, critical pertussis remains difficult to treat.

## Figures and Tables

**Table 1:** Demographics and illness history prior to PICU admission by survival, n (%)

	Overall N=17	Survivors N=13	Deaths N=4

**Demographics**			
Women, n (%)	9 (52)	6 (46)	3 (75)
Median Age (days);IQR	56 (24–630)	36 (24–630)	88,5(80–184)
Age, n (%)			
<2 months	9	9	0
2–12 months	7	3	4
1 year or older	1	1	0
Co-morbidity, n (%)	7(41)	6(46)	1(25)
Premature	5(29)	4(30)	1(25)
Small for gestational age at birth	1(6)	1(8)	0
Encephalopathy	1(6)	1(8)	0
Immunization, n (%)	5(29)	1(7.6)	4(100)

**History of current illness, n (%)**			
fever	7(41)	4(30)	3(75)
Cough started relative to PICU admission:			
< 1 wk prior	5(29)	4(30)	1(25)
1–2 wk prior	4(23)	3(23)	1(25)
≥2 wk prior	8(47)	7(54)	2(50)
Respiratory distress/hard to catch breath	11(65)	8(61)	3(75)
Cyanotic coughing	16(94)	13(100)	3(75)
Apnea	3(18)	3(23)	0
Seizures	1(6)	0	1(25)
